# P48 Process evaluation of an intervention to optimize antibiotic use in dentistry

**DOI:** 10.1093/jacamr/dlaf118.055

**Published:** 2025-07-14

**Authors:** Wendy Thompson, Vanessa Carter, Laurie Powell, Tanya Walsh, Lucie Byrne-Davis

**Affiliations:** University of Manchester, Manchester, UK; WHO AMR Survivor Group, Geneva, Switzerland; University of Manchester, Manchester, UK; University of Manchester, Manchester, UK; University of Manchester, Manchester, UK

## Abstract

**Background:**

Antibiotic resistance is driven by antibiotic use. Antibiotic use is common for toothache and dental infection. In 2023, 2.5 million of the 36 million antibiotic prescriptions across England’s National Health Service were from dentists. Toothache is often caused by inflammation rather than infection. Most toothaches and dental infections are readily amenable to dental procedures, such as root canal treatment, without needing antibiotics. Antibiotics are only necessary with signs of infection spreading systemically, for example sepsis. Previous studies have shown up only around 20% of antibiotics prescribed by NHS dentists were strictly necessary, in accordance with clinical guidelines. An evidence-based, behaviour-science informed antibiotic stewardship intervention to support shared-decision making during urgent dental appointments has been developed. The intervention is a leaflet designed for use by dentists and patients at point-of-care, together with motivational training for dentists.

**Objectives:**

To test acceptability among dentists of the intervention; to seek a signal of its effectiveness at optimizing antibiotic prescribing; and to evaluate how the intervention worked in practice.

**Methods:**

In 2023, general dentists were randomized to provide care with or without the intervention during their regular urgent dental care appointments. Dentists in the intervention group received 1 h of on-line training to use the leaflet to assist communication with patients at point-of-care. After treating two patients, each participating dentist completed an on-line survey via the Qualtrics^™^ platform. The on-line survey included a mix of qualitative questions about acceptability of the intervention to the dentists, and quantitative questions relating to elements of the international Core Outcome Set for Dental Antibiotic Stewardship: quantity of antibiotics prescribed, and adverse outcomes reported. Process evaluation questions were based on the six-item brief measure of capabilities, opportunities and motivation (COM-B), adapted from Keyworth et al (2020). Analysis of the qualitative questions used thematic analysis. Mean scores for each COM-B component and descriptive statistics were used to report the quantitative data.

**Results:**

Dentists felt that the intervention was ‘quick, easy and useful’ with ‘no increase in time taken’ and ‘having the leaflet really helped when explaining.’ Dentists (*n*=8) using the intervention with patients (*n*=16) prescribed two antibiotics. Of these, one antibiotic was prescribed by a dentist who received training but did not use the intervention at point-of-care. Dentists (*n*=9) who did not use the intervention with patients (*n*=18) recorded four antibiotic prescriptions. One dentist in this group did not report whether they prescribed any antibiotics to either of their two patients. No adverse outcomes were recorded in either group. All six COM-B components scored better when the intervention was used. Whilst the greatest improvement was seen in physical opportunity, this component had the lowest score in both arms of the study.

**Conclusions:**

The intervention is acceptable to dentists. The change in COM-B components and differences in prescribing between intervention and control groups provide a signal that it might work. Given the promising results from this feasibility study, the intervention is worthy of further exploration in a clinical trial to fully test its effectiveness at helping optimize dental antibiotic prescribing.

